# The First Application of a Gd_3_Al_2_Ga_3_O_12_:Ce Single-Crystal Scintillator to Neutron Radiography

**DOI:** 10.3390/jimaging7110232

**Published:** 2021-11-02

**Authors:** Kazuhisa Isegawa, Daigo Setoyama, Hidehiko Kimura, Takenao Shinohara

**Affiliations:** 1 Japan Atomic Energy Agency, Tokai 319-1195, Japan; takenao.shinohara@j-parc.jp; 2 Toyota Central R&D Laboratories, Nagakute 480-1192, Japan; daigo@mosk.tytlabs.co.jp (D.S.); hdkimura@mosk.tytlabs.co.jp (H.K.)

**Keywords:** neutron radiography, neutron imaging, neutron scintillator, gadolinium aluminium gallium garnet

## Abstract

Neutron radiography is regarded as complementary to X-ray radiography in terms of transmittance through materials, but its spatial resolution is still insufficient. In order to achieve higher resolution in neutron imaging, several approaches have been adopted, such as optical magnification and event centroiding. In this paper, the authors focused on modification of the scintillator. A Gd_3_Al_2_Ga_3_O_12_:Ce single-crystal scintillator was applied to neutron radiography for the first time and a spatial resolution of 10.5 μm was achieved. The results indicate that this material can be a powerful candidate for a new neutron scintillator providing a resolution in micrometer order by optimizing the optical system and increasing the scintillator luminosity.

## 1. Introduction

Neutron radiography is a nondestructive method to observe the inside of samples. X-ray radiography is a similar and better-known technique but complementary in principle to neutron radiography. The X-ray cross section of each element is almost proportional to the squared atomic number, whereas for neutrons, it shows complex relationships. For example, neutrons penetrate metal structures but not hydrogen and lithium, so neutron radiography is suitable for the observation of fuel cells and Li-ion batteries [1,2]. However, the spatial resolution of neutron radiography is several orders of magnitude lower than that of X-ray radiography in terms of the maximum achievable value. Realization of both high resolution and high brightness expands the application range of neutron radiography. For example, the elemental distribution and behavior inside a multi-material sample composed of resins and metals can be observed at the same micrometer order.

Many demands exist for high-spatial-resolution neutron radiography of less than 10 μm, and various approaches are being developed to improve the resolution of neutron radiography: optical magnification [3,4,5], event centroiding [6,7,8], track recording [9,10], and neutron focusing techniques [11]. Optical magnification systems have been recently developed by the Paul Scherrer Institut (PSI) group, achieving resolutions of 10 μm by a fiber optics taper [3] and 5 μm by a neutron microscope [4,5]. The event centroiding process has been conducted at ISIS, LANSCE, and NIST [6,7,8]. As a result, a very high resolution of 2 μm has been achieved at NIST [8].

On the other hand, exploration of high-performance scintillators can also be an approach to achieve higher resolution imaging. The optical characteristics of scintillators also greatly affect the quality of obtained images because, in many cases, the spread of the bright points in the scintillator initially defines the resolution. Several types of powder scintillators, for example, ^6^LiF/ZnS and gadolinium oxysulfide, Gd_2_O_2_S:Tb, named P43 or Gadox, have been mostly used for neutron radiography. The resolution and luminosity of the powder scintillators are in a trade-off relationship, that is, a thicker scintillator plate brings about a higher luminosity, whereas the resolution becomes lower due to photon scattering inside the scintillator [12]. Consequently, most high-resolution imaging employs a Gadox scintillator enriched with the ^157^Gd isotope because it exhibits the largest absorption cross section for low-energy neutrons and, hence, it is possible to make a very thin scintillator [3,4,5,13]. We have been interested in another promising candidate, a transparent single-crystal scintillator composed of elements with large neutron absorption cross sections, which is expected to provide both high resolution and high luminosity due to smaller photon scattering and attenuation inside than those for powder scintillator materials, irrespective of its thickness [12].

Previously, the Helmholtz-Zentrum Berlin group tested a transparent Gd_3_Ga_5_O_12_:Eu (GGG) single-crystal scintillator [14], but no subsequent studies have been reported after that. Recently, a single-crystal scintillator, Gd_3_Al_2_Ga_3_O_12_:Ce (GAGG), has been developed by Yoshikawa and coworkers [15,16], and it is widely utilized for X-ray radiography because of its high luminosity and fast decay time for X-rays [17]. Because GAGG contains a high amount of Gd atoms like GGG and shows good optical transparency for the emitted light with a wavelength of 520 nm, it is expected to be utilized for high-resolution neutron-imaging applications. The decay time of GAGG is 0.2 μs [17], while that of GGG is 800 μs [18]. The short decay time of GAGG is very suitable for the energy-resolved neutron imaging using the pulsed neutron beam, in which the neutron energy is deduced by the time-of-flight method and a fine temporal resolution is required. However, the application of a GAGG single crystal to neutron detection is very limited [19], and, to our knowledge, no previous neutron-imaging study using it exists.

In this study, we applied a transparent single-crystal scintillator GAGG to neutron imaging for the first time, and evaluated the performance and potential of GAGG by comparing neutron images obtained using GAGGs with different thicknesses and a P43 scintillator.

## 2. Materials and Methods

### 2.1. Neutron-Imaging System and Experimental Conditions

**Figure 1:**
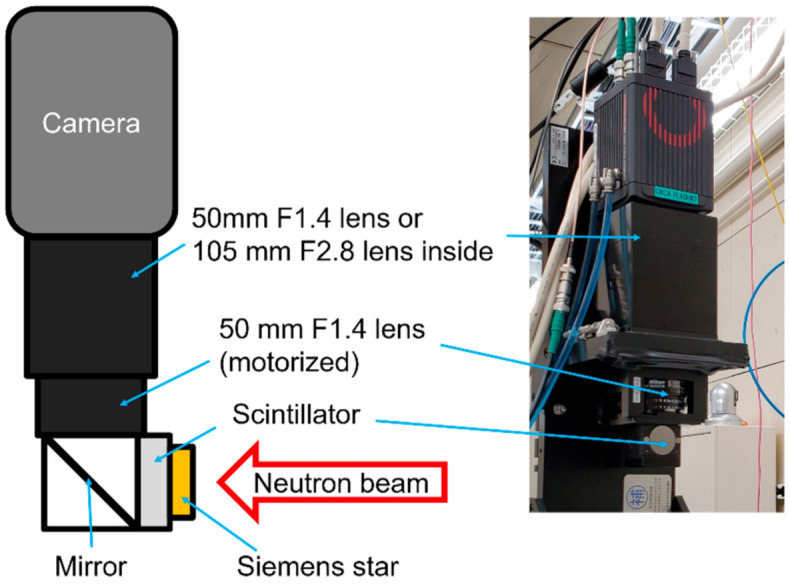
Schematic and picture of the experimental setup.

Experiments were performed at the energy-resolved neutron-imaging system RADEN, which was installed at beamline number 22 (BL22) in the Materials and Life Science Experimental Facility (MLF) of the Japan Proton Accelerator Research Complex (J-PARC) [20]. This instrument is equipped with several neutron detectors and have achieved a minimum spatial resolution of 30 μm using a ^6^LiF/ZnS scintillator and a cooled Charge Coupled Device (CCD) camera. For this study, we newly prepared an imaging system, which was originally designed for X-ray imaging with a spatial resolution of less than 10 µm. A schematic of the imaging system is shown in Figure 1. This system consisted of a Complementary Metal–Oxide–Semiconductor (CMOS) camera, two optical lenses, a mirror, and a scintillator. The CMOS camera is an ORCA-Flash4.0 V3 of Hamamatsu Photonics K.K. (Hamamatsu, Japan) with 2048 × 2048 pixels of 6.5 μm square sensors. The two lenses composed an infinity-corrected optical system [13,14]. The primary lens was 50 mm F1.4 from Nikon (Tokyo, Japan), and, for the secondary lens, we used a 50 mm F1.4 lens and a 105 mm F2.8 lens, both from Nikon, to change the image magnification to 1.0 and 2.1, respectively. These lens conditions are referred to as the “1×” and “2×” conditions hereafter. The fields of view (FoV) are 13.3 mm square and 6.6 mm square, respectively. Two GAGG scintillators, which were commercial products for X-ray imaging, with thicknesses of 10 and 100 µm, were adopted in the experiment, and a P43 with 10 µm thickness was also used for comparison. The diameter of the GAGG scintillator was 15 mm and it was supported by a 1 mm-thick amorphous carbon backplate, while that of the P43 screen was 16 mm, whose substrate was a 0.5 mm-thick beryllium plate. All scintillators were purchased from Hamamatsu Photonics K.K. A Siemens star target made of Gd thin film evaporated onto a quartz substrate from PSI, whose inner line pair was 20 μm wide and whose outer was 500 μm wide [21], was used to evaluate the spatial resolution performance. This target was directly attached to a support plate of a scintillator to minimize geometrical blurring.

The 1× experiments were conducted at the far sample position of RADEN located 23 m away from the source. The *L*/*D* values, which were related to the beam divergence and, hence, to the spatial resolution, defined by the distance *L* between the aperture and the sample and the aperture diameter *D* were 230 and 400. The expected neutron fluxes for *L*/*D* of 230 and 400 were 1.0 × 10^7^ and 3.4 × 10^6^ n/s/cm^2^, respectively. In contrast, the 2× experiments were conducted at the near sample position, which was 18 m away from the source, and the *L*/*D* values were 180 and 300, respectively. This is because approaching the source allows us to select a smaller *L*/*D*, which provides larger neutron flux, and compensates for the decrease of effective neutron intensity according to the decrease of the FoV by 2× image magnification. The neutron fluxes for *L*/*D* of 180 and 300 were 1.7 × 10^7^ and 6.1 × 10^6^ n/s/cm^2^, respectively. The accumulation times were 10 s × 90 times for *L*/*D* = 180 and 230 and 10 s × 180 times for *L*/*D* = 300 and 400. For suppression of the white spots in the obtained images, median filtering was applied on the accumulated images but not within the plane of the image. Then, the transmission image was obtained by dark current correction and open-beam normalization. For both experiments, the neutron wavelength range was adjusted from 1.50 to 6.48 Å by using a disk chopper.

### 2.2. Methods for Calculating Spatial Resolution

The spatial resolution of the images was calculated on the basis of the modulator transfer function (MTF). MTF is obtained by the Fourier transform of the line spread function (LSF), which is the derivative of the edge spread function (ESF). By selecting the fitting function appropriately, the resolution can be analytically calculated with parameters from fitting the ESF. Various types of unsharpness caused by sample vibration, neutron diffusion, scintillator, camera, and so on are expressed as unique functions [22,23,24]. LSF is generally approximated as Gaussian by convolution of functions of all unsharpness on the basis of the central limit theorem [22,23]. In high-resolution imaging, the unsharpness of the scintillator represented as Lorentzian affects the total unsharpness significantly [23]. Therefore, we adopted the Voigt function, which is a convolution of Gaussian and Lorentzian, as the fitting function. The integral form of the Voigt function was used to fit the ESFs as:

(1) Vx=Re12+12erfzx+izx2πF221,1;32,2;−zx2.

The variable *z*(*x*) is expressed as:

(2) $$z\left(x\right)=\frac{\sqrt{\pi }}{Bw\left(i\rho \right)}\left(x-\mu \right)+i\rho .$$

_2_F_2_ is a generalized hypergeometric function, and *w*(*x*) is the Faddeeva function. The parameters are defined as follows: *i* is the imaginary unit, *μ* is the inflection point, *B* is the integral width, and *ρ* is the Lorentzian contribution. With the fitting parameters and the conversion factor *d* (μm/pixel), the 10% MTF resolution *δ_MTF_* can be represented as:

(3) $$\delta_{MTF}=\frac{d}{2}\frac{\sqrt{\pi }Bw\left(i\rho \right)}{\left(-\rho +\sqrt{\rho^{2}+\ln10}\right)}.$$

In this study, *d* was defined by dividing the element size of the CMOS camera (6.5 μm) by the image magnification. It should be noted that the MTF-based resolution was originally defined as per line pair [7,14] but we applied the resolution per one line to obtain values close to visual observation according to a previous paper [6].

**Figure 2:**
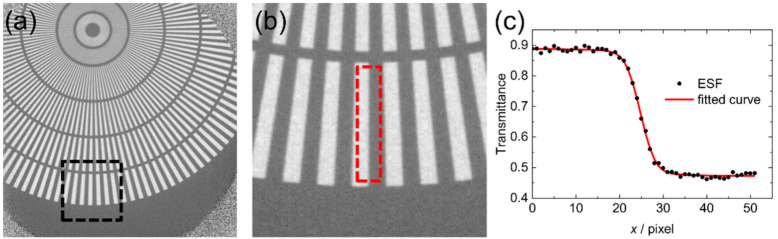
Process of obtaining ESF from a neutron transmission image: (**a**) whole image example, (**b**) enlargement of the dashed area in (**a**,**c**) ESF of the dashed area in (**b**) and fitting curve.

As shown in Figure 2a, in order to average over a sufficiently large area, ESFs were obtained from the outer shell of the Siemens star. Then, the resolutions were calculated from the fitting parameters of ESFs by the method in this section.

## 3. Results and Discussion

**Figure 3:**
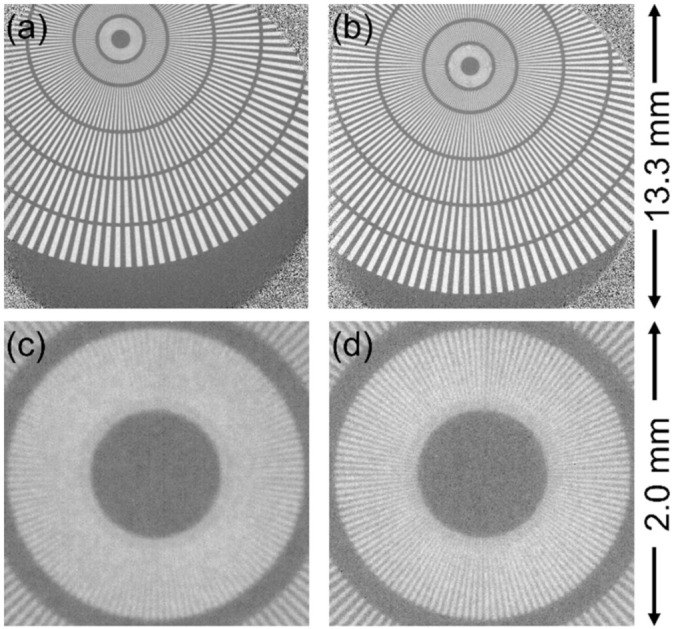
Neutron transmission images taken at 1× magnification condition with *L*/*D* = 400. (**a**) An image taken with P43 (10 μm), (**b**) an image taken with GAGG (100 μm). (**c**) and (**d**) are enlarged images of (**a**) and (**b**), respectively.

The performance of each scintillator was evaluated by using the Siemens star target with the optical magnification and *L*/*D* as variables. First, the 1× experiments using the 50 mm F1.4 lens for the secondary lens were conducted with GAGG (100 μm) and P43 (10 μm) scintillators. The obtained transmission images are shown in Figure 3. Because the scintillator could not cover the full area of the FoV in the 1× condition, the four corners of the image did not contribute to the neutron transmission image. Although 20 μm-wide line pairs at the center of the Siemens star pattern were not distinguished with either scintillator, as seen in Figure 3, the inner line pairs were more distinguishable by using the GAGG than when by using the P43 scintillator. This was demonstrated by the evaluated MTF values, shown in Table 1. Thus, this experimental result shows that the GAGG could achieve a better spatial resolution than the P43, despite the 10-times-larger thickness, which was a confirmation of the superior performance of the transparent single-crystal scintillator. The change in MTF values for different *L*/*D* is discussed later.

**Figure 4:**
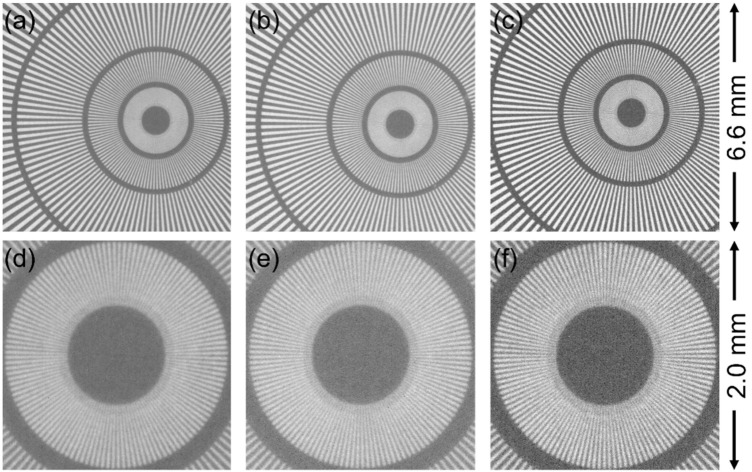
High-resolution neutron radiography taken at 2× magnification under *L*/*D* = 300 conditions by three types of scintillators: (**a**) an image taken with P43 (10 μm), (**b**) an image taken with GAGG (100 μm), (**c**) an image taken with GAGG (10 μm), (**d**) an enlarged view of (**a**), (**e**) an enlarged view of (**b**), and (**f**) an enlarged view of (**c**).

Next, the 2× experiments using the 105 mm F2.8 lens for the secondary lens were conducted. The entire FoV was within the effective area of scintillators in this condition. According to the results of the 1× condition that the GAGG scintillator was confirmed to produce enough light by neutrons, we also employed a 10-µm-thick GAGG scintillator anticipating a higher spatial resolution than the 100-µm-thick one in addition to the scintillators used in the former experiments. Figure 4 shows the neutron transmission images at *L*/*D* = 300 in the 2× condition for three scintillators. The 10-μm-wide lines in the center of the Siemens star pattern were successfully distinguished by using all scintillators. The image taken with the GAGG (100 μm), shown in Figure 4b,e, appeared as clear as that taken with the P43 scintillator (Figure 4a,d), similar to the results of the 1× condition. By using the 10-µm-thick GAGG scintillator, which should cause the lowest photon scattering and attenuation, the contrast of the image appeared to be further enhanced, as seen in Figure 4c,f. Actually, the Michelson contrast of P43, 100-µm-thick GAGG, and 10-µm-thick GAGG was calculated to be 45%, 44%, and 57%, respectively.

The resolution derived from Figure 4 is summarized in Table 1. The best resolution of 10.5 μm was achieved by using the GAGG (100 μm) at 2× magnification under *L*/*D* = 300 condition. Despite the thinness, the 10-µm-thick GAGG scintillator did not achieve the highest spatial resolution. The reasons for this inferior performance compared to the thicker GAGG are nonuniformity of the image and difficulty in focus adjustment due to the small light yield.

Here, we will mention the difference in MTF-based resolution with the different *L*/*D*s. In general, a large *L*/*D* decreases neutron flux but allows access to a fine spatial resolution. This characteristic could be found in the study using GAGG scintillators, and higher resolutions could be obtained with larger *L*/*D*s. Conversely, the results with the P43 scintillator did not follow this rule. The reason is not clear, but may be due to the insufficient focus adjustment, and hence, a failure to correctly recognize the difference in MTF values. The precise focus adjustment is essential for achieving higher resolution and for a fair comparison among the scintillators. However, the fact that the 100-µm-thick GAGG scintillator could achieve 10 µm resolution still strongly supports the superior performance of the transparent single-crystal scintillator.

**Figure 5:**
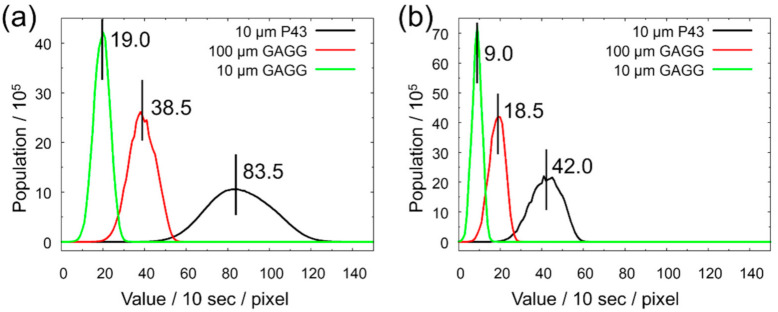
Histograms of open-beam intensity taken at 2× magnification with scintillators P43 (10 μm), GAGG (100 μm), and GAGG (10 μm): (**a**) *L*/*D* = 180 and (**b**) *L*/*D* = 300. The position and value of the median are shown in the figure.

The effective luminance histograms per 10 s for each scintillator, which were calculated by subtracting the background luminance from the direct beam luminance of 2× experiments, are shown in Figure 5. This figure indicates that the GAGG scintillator with 100 μm thickness was about half as luminant as the P43, and the GAGG with 10 µm thickness was half of that. From the composition of each scintillator, the attenuation coefficients for thermal neutrons can be calculated to be 1157.0 cm^−1^ and 642.1 cm^−1^ for P43 and GAGG, respectively. Then, the thermal neutron transmission was assumed to be 31.4% for the 10-µm-thick P43, 0.162% for the 100-µm-thick GAGG, and 52.0% for the 10-µm-thick GAGG scintillator. Hence, the different effective luminance between GAGGs can be easily understood from the different amounts of the absorbed neutrons. This also implies that the self-attenuation of emitted light was negligibly small. Considering the effective luminance normalized with the neutron attenuation rate, the P43 was three times brighter than the GAGG. Although the light emission performance of GAGG for neutron irradiation was expected to be comparable to that of P43 considering the results of X-ray irradiation [17,25], it was revealed that there was a large difference between the two scintillators in reality.

Finally, we discuss the prospects of the high-spatial-resolution neutron imaging with the GAGG scintillator. The current performance of the GAGG does not fully surpass that of the P43. However, the GAGG still has room to improve its performance as a neutron scintillator because the GAGG used in this study is not optimized for neutron application but for X-ray experiments. The decay time of emitted light is too short, and considering the X-ray energy resolution is not necessary. Therefore, material design to increase the light yield by neutron irradiation will make the GAGG a good neutron scintillator, for example, by selecting doping elements and optimizing their amounts to generate suitable emission centers similar to studies conducted for X-ray use [26,27,28], or adjusting matrix crystal composition to exhibit an appropriate band gap and high enough Gd concentration. Owing to increase in the light yield, the GAGG will be a good alternative candidate to Gadox enriched with ^157^Gd isotope. Because of its transparency and small light scattering, the GAGG can be made thicker to increase the efficiency without degrading the resolution. Then, both scintillators will produce comparable luminance. Because the GAGG does not need to be enriched with isotopes, it can reduce the cost substantially. Moreover, a newly developed 4-inch GAGG single crystal will enable imaging with a larger FoV [29]. Those will make a significant contribution to conducting high-resolution neutron imaging more easily.

## 4. Conclusions

The transparent single-crystal GAGG scintillator, which was developed for X-ray radiography, was used in neutron radiography for the first time and achieved a spatial resolution of 10.5 μm. The 100-μm-thick GAGG scintillator showed higher spatial resolution than that of the 10-μm-thick P43 powder scintillator because transparent single-crystal scintillators reduce internal scattering and attenuation of light regardless of thickness. These results provide potential for an inexpensive high-resolution scintillator without a ^157^Gd isotope. By optimizing the composition of GAGG to the neutron use, high-resolution neutron imaging will be more common.

## Figures and Tables

**Table 1:** Summary of the spatial resolution of neutron radiography of the Siemens star test pattern.

	Spatial Resolution/μm			
Magnification	1×		2×	
FoV/mm^2^	13.3 × 13.3		6.6 × 6.6	
*L*/*D*	230	400	180	300
P43 (10 μm)	23.8	25.5	12.9	13.8
GAGG (100 μm)	16.6	15.0	13.0	10.5
GAGG (10 μm)			12.6	11.7

## Data Availability

Not applicable.
